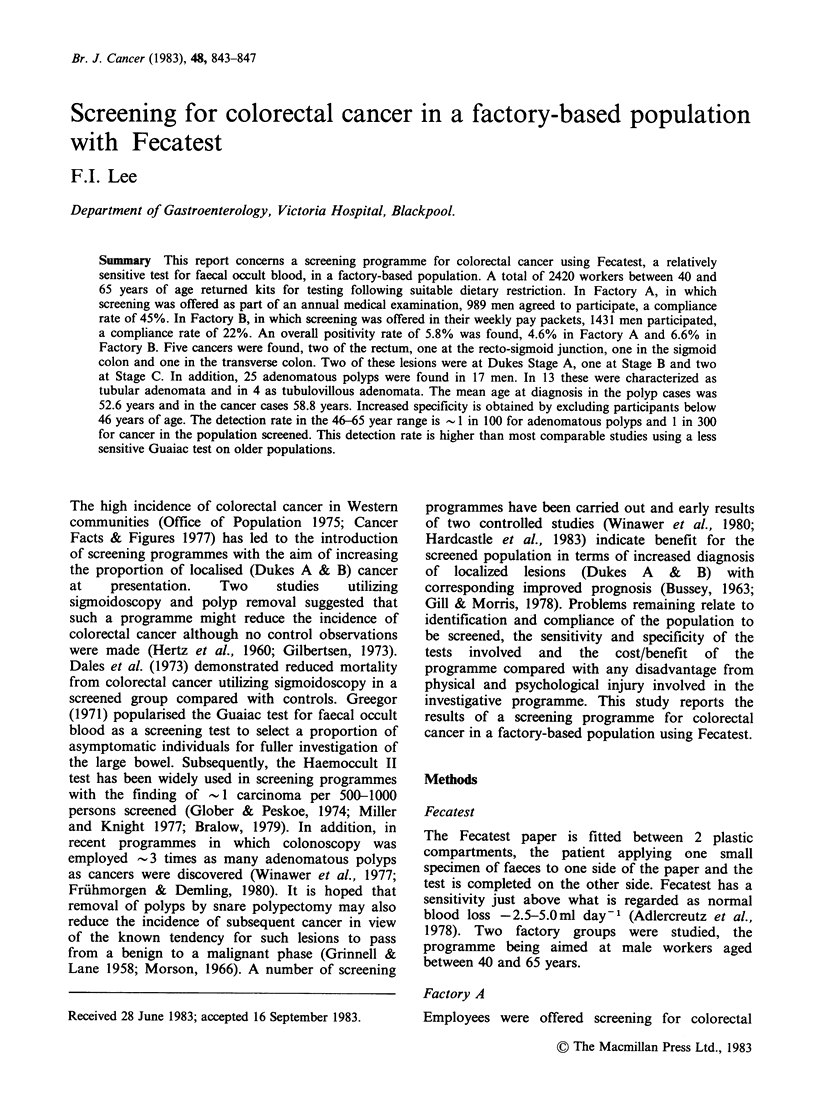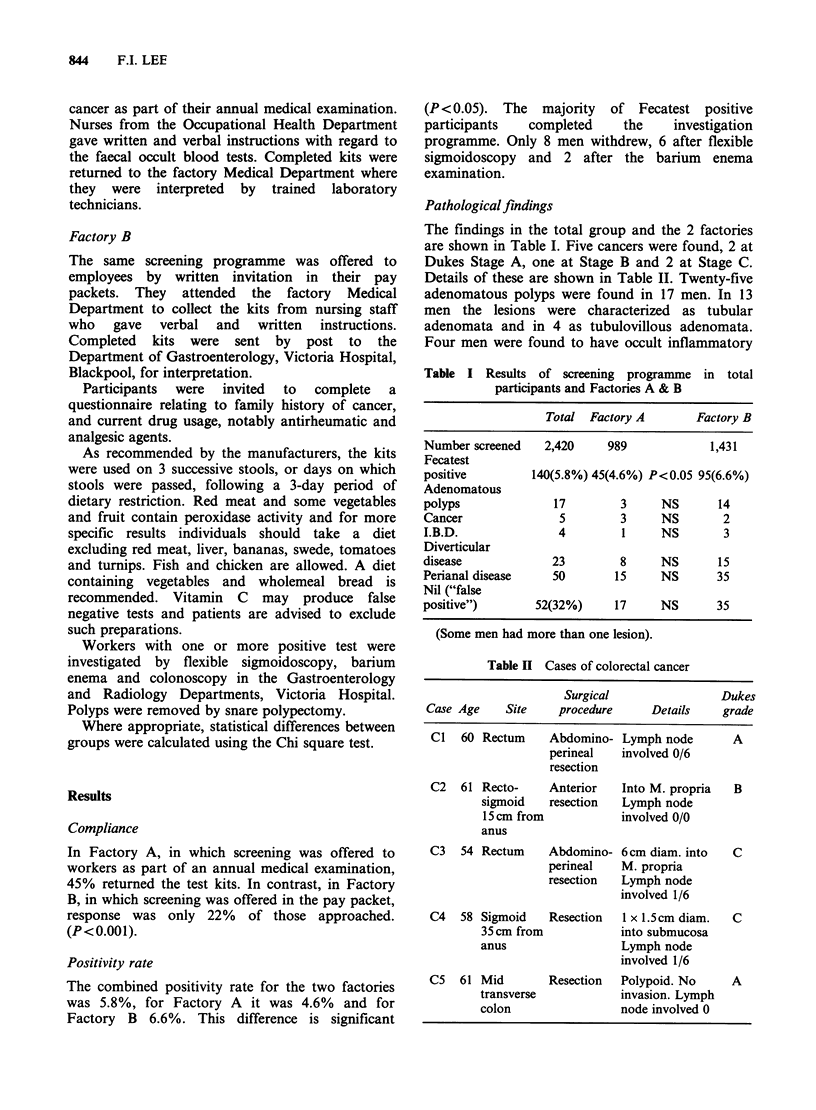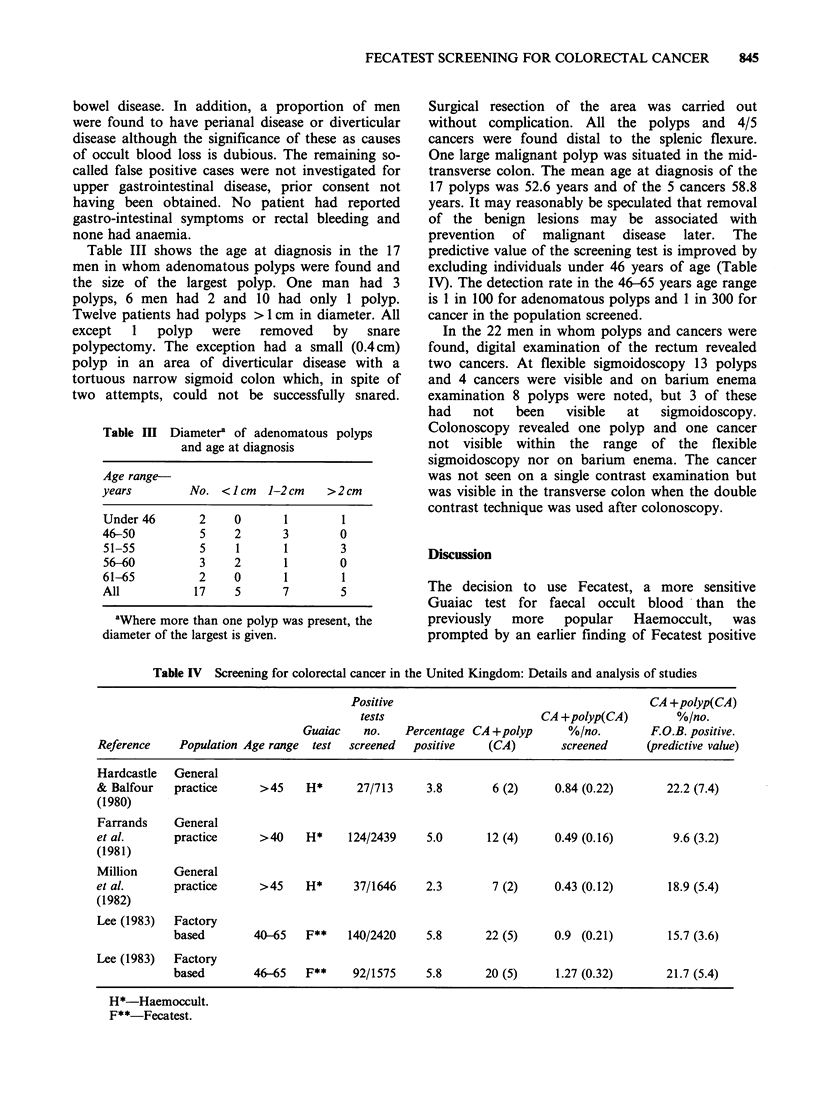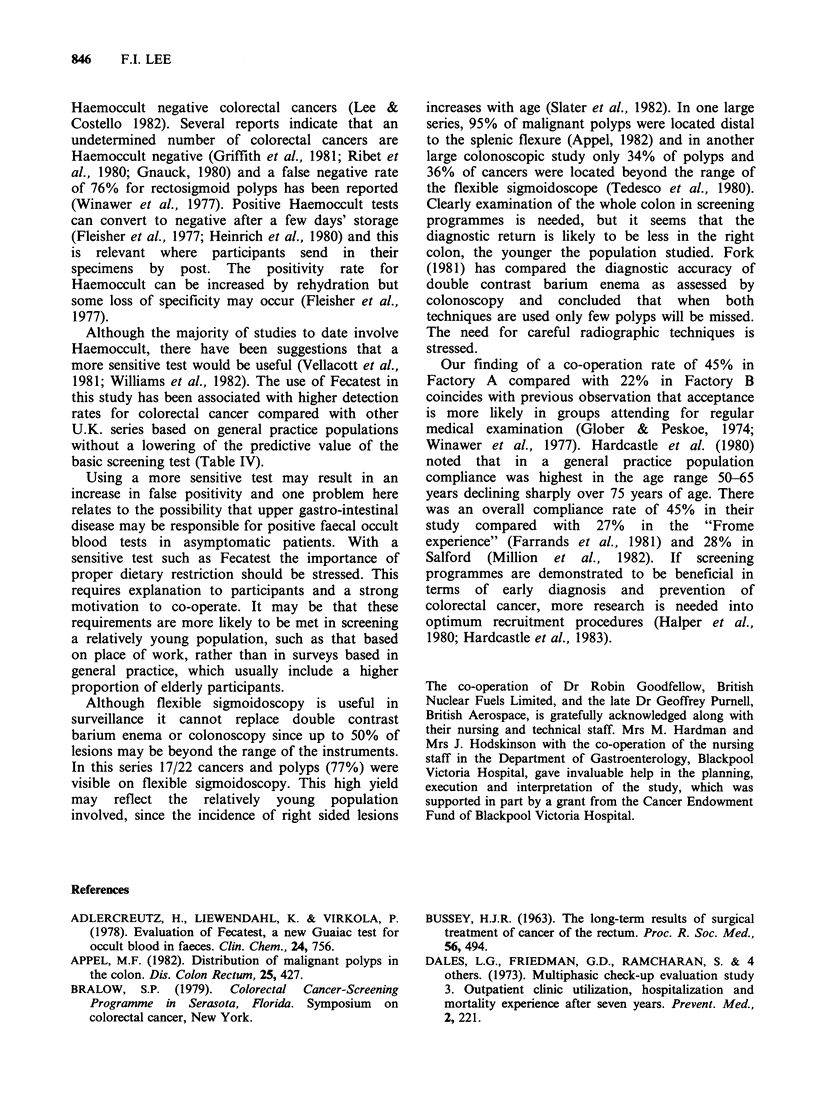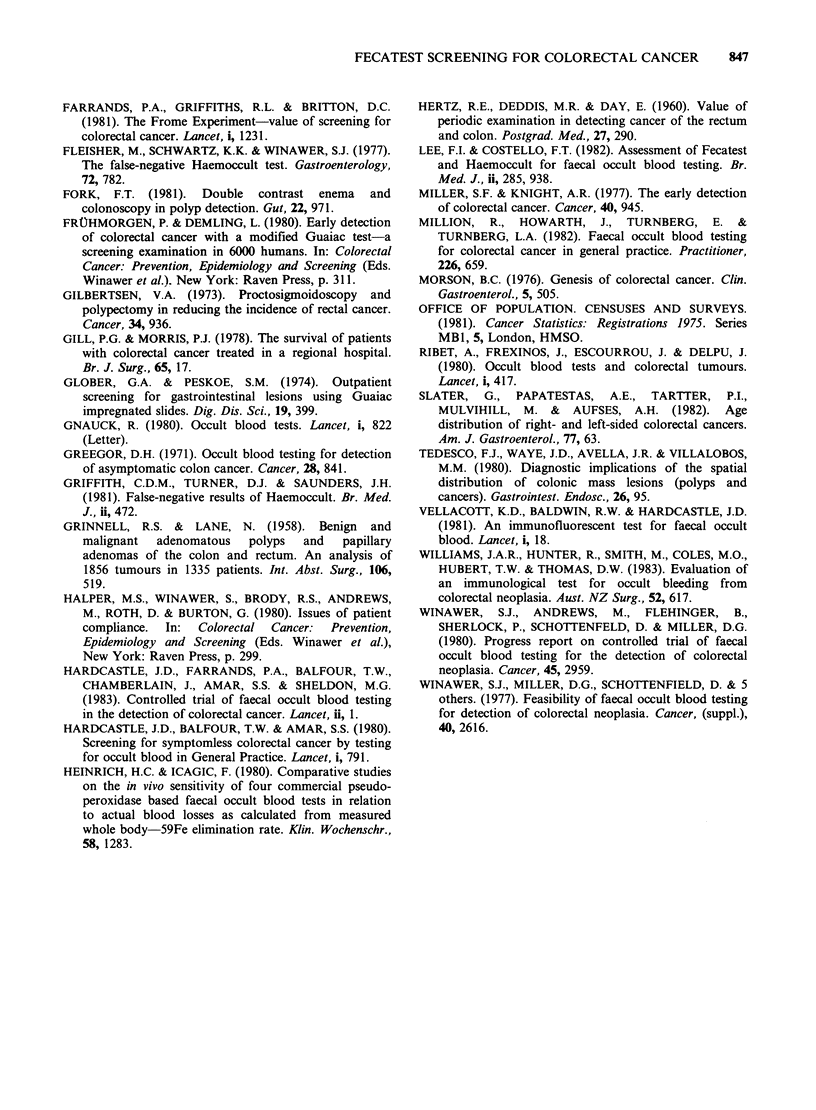# Screening for colorectal cancer in a factory-based population with Fecatest.

**DOI:** 10.1038/bjc.1983.275

**Published:** 1983-12

**Authors:** F. I. Lee

## Abstract

This report concerns a screening programme for colorectal cancer using Fecatest, a relatively sensitive test for faecal occult blood, in a factor-based population. A total of 2420 workers between 40 and 65 years of age returned kits for testing following suitable dietary restriction. In Factory A, in which screening was offered as part of an annual medical examination, 989 men agreed to participate, a compliance rate of 45%. In Factory B, in which screening was offered in their weekly pay packets, 1431 men participated, a compliance rate of 22%. An overall positivity rate of 5.8% was found, 4.6% in Factory A and 6.6% in Factory B. Five cancers were found, two of the rectum, one at the recto-sigmoid junction, one in the sigmoid colon and one in the transverse colon. Two of these lesions were at Dukes Stage A, one at Stage B and two at Stage C. In addition, 25 adenomatous polyps were found in 17 men. In 13 these were characterized as tubular adenomata and in 4 as tubulovillous adenomata. The mean age at diagnosis in the polyp cases was 52.6 years and in the cancer cases 58.8 years. Increased specificity is obtained by excluding participants below 46 years of age. The detection rate in the 46-65 year range is approximately 1 in 100 for adenomatous polyps and 1 in 300 for cancer in the population screened. This detection rate is higher than most comparable studies using a less sensitive Guaiac test on older populations.


*Br. J. Cancer* (1983), **48**, 843–847

### Screening for colorectal cancer in a factory-based population with Fecatest

F.I. Lee

*Department of Gastroenterology, Victoria Hospital, Blackpool.*

**Summary** This report concerns a screening programme for colorectal cancer using Fecatest, a relatively sensitive test for faecal occult blood, in a factory-based population. A total of 2420 workers between 40 and 65 years of age returned kits for testing following suitable dietary restriction. In Factory A, in which screening was offered as part of an annual medical examination, 989 men agreed to participate, a compliance rate of 45%. In Factory B, in which screening was offered in their weekly pay packets, 1431 men participated, a compliance rate of 22%. An overall positivity rate of 5.8% was found, 4.6% in Factory A and 6.6% in Factory B. Five cancers were found, two of the rectum, one at the recto-sigmoid junction, one in the sigmoid colon and one in the transverse colon. Two of these lesions were at Dukes Stage A, one at Stage B and two at Stage C. In addition, 25 adenomatous polyps were found in 17 men. In 13 these were characterized as tubular adenomata and in 4 as tubulovillous adenomata. The mean age at diagnosis in the polyp cases was 52.6 years and in the cancer cases 58.8 years. Increased specificity is obtained by excluding participants below 46 years of age. The detection rate in the 46–65 year range is ~1 in 100 for adenomatous polyps and 1 in 300 for cancer in the population screened. This detection rate is higher than most comparable studies using a less sensitive Guaiac test on older populations.

The high incidence of colorectal cancer in Western communities (Office of Population 1975; Cancer Facts & Figures 1977) has led to the introduction of screening programmes with the aim of increasing the proportion of localised (Dukes A & B) cancer at presentation. Two studies utilizing sigmoidoscopy and polyp removal suggested that such a programme might reduce the incidence of colorectal cancer although no control observations were made (Hertz *et al.*, 1960; Gilbertsen, 1973). Dales *et al.* (1973) demonstrated reduced mortality from colorectal cancer utilizing sigmoidoscopy in a screened group compared with controls. Greegor (1971) popularised the Guaiac test for faecal occult blood as a screening test to select a proportion of asymptomatic individuals for fuller investigation of the large bowel. Subsequently, the Haemoccult II test has been widely used in screening programmes with the finding of ~1 carcinoma per 500–1000 persons screened (Glober & Peskoe, 1974; Miller and Knight 1977; Bralow, 1979). In addition, in recent programmes in which colonoscopy was employed ~3 times as many adenomatous polyps as cancers were discovered (Winawer *et al.*, 1977; Frühmorgen & Demling, 1980). It is hoped that removal of polyps by snare polypectomy may also reduce the incidence of subsequent cancer in view of the known tendency for such lesions to pass from a benign to a malignant phase (Grinnell & Lane 1958; Morson, 1966). A number of screening programmes have been carried out and early results of two controlled studies (Winawer *et al.*, 1980; Hardcastle *et al.*, 1983) indicate benefit for the screened population in terms of increased diagnosis of localized lesions (Dukes A & B) with corresponding improved prognosis (Bussey, 1963; Gill & Morris, 1978). Problems remaining relate to identification and compliance of the population to be screened, the sensitivity and specificity of the tests involved and the cost/benefit of the programme compared with any disadvantage from physical and psychological injury involved in the investigative programme. This study reports the results of a screening programme for colorectal cancer in a factory-based population using Fecatest.

### Methods

### *Fecatest*

The Fecatest paper is fitted between 2 plastic compartments, the patient applying one small specimen of faeces to one side of the paper and the test is completed on the other side. Fecatest has a sensitivity just above what is regarded as normal blood loss $-2.5$–$5.0\,\mathrm{ml\ day^{-1}}$ (Adlercreutz *et al.*, 1978). Two factory groups were studied, the programme being aimed at male workers aged between 40 and 65 years.

### *Factory A*

Employees were offered screening for colorectal

Received 28 June 1983; accepted 16 September 1983.

© The Macmillan Press Ltd., 1983

cancer as part of their annual medical examination. Nurses from the Occupational Health Department gave written and verbal instructions with regard to the faecal occult blood tests. Completed kits were returned to the factory Medical Department where they were interpreted by trained laboratory technicians.

*Factory B*

The same screening programme was offered to employees by written invitation in their pay packets. They attended the factory Medical Department to collect the kits from nursing staff who gave verbal and written instructions. Completed kits were sent by post to the Department of Gastroenterology, Victoria Hospital, Blackpool, for interpretation.

Participants were invited to complete a questionnaire relating to family history of cancer, and current drug usage, notably antirheumatic and analgesic agents.

As recommended by the manufacturers, the kits were used on 3 successive stools, or days on which stools were passed, following a 3-day period of dietary restriction. Red meat and some vegetables and fruit contain peroxidase activity and for more specific results individuals should take a diet excluding red meat, liver, bananas, swede, tomatoes and turnips. Fish and chicken are allowed. A diet containing vegetables and wholemeal bread is recommended. Vitamin C may produce false negative tests and patients are advised to exclude such preparations.

Workers with one or more positive test were investigated by flexible sigmoidoscopy, barium enema and colonoscopy in the Gastroenterology and Radiology Departments, Victoria Hospital. Polyps were removed by snare polypectomy.

Where appropriate, statistical differences between groups were calculated using the Chi square test.

### Results

*Compliance*

In Factory A, in which screening was offered to workers as part of an annual medical examination, 45% returned the test kits. In contrast, in Factory B, in which screening was offered in the pay packet, response was only 22% of those approached. ($P<0.001$).

*Positivity rate*

The combined positivity rate for the two factories was 5.8%, for Factory A it was 4.6% and for Factory B 6.6%. This difference is significant ($P<0.05$). The majority of Fecatest positive participants completed the investigation programme. Only 8 men withdrew, 6 after flexible sigmoidoscopy and 2 after the barium enema examination.

*Pathological findings*

The findings in the total group and the 2 factories are shown in Table I. Five cancers were found, 2 at Dukes Stage A, one at Stage B and 2 at Stage C. Details of these are shown in Table II. Twenty-five adenomatous polyps were found in 17 men. In 13 men the lesions were characterized as tubular adenomata and in 4 as tubulovillous adenomata. Four men were found to have occult inflammatory

**Table I** Results of screening programme in total participants and Factories A & B

| | *Total* | *Factory A* | | *Factory B* |
|---|---|---|---|---|
| Number screened | 2,420 | 989 | | 1,431 |
| Fecatest positive | 140(5.8%) | 45(4.6%) | $P<0.05$ | 95(6.6%) |
| Adenomatous polyps | 17 | 3 | NS | 14 |
| Cancer | 5 | 3 | NS | 2 |
| I.B.D. | 4 | 1 | NS | 3 |
| Diverticular disease | 23 | 8 | NS | 15 |
| Perianal disease | 50 | 15 | NS | 35 |
| Nil ("false positive") | 52(32%) | 17 | NS | 35 |

(Some men had more than one lesion).

**Table II** Cases of colorectal cancer

| *Case* | *Age* | *Site* | *Surgical procedure* | *Details* | *Dukes grade* |
|---|---|---|---|---|---|
| C1 | 60 | Rectum | Abdomino-perineal resection | Lymph node involved 0/6 | A |
| C2 | 61 | Recto-sigmoid 15 cm from anus | Anterior resection | Into M. propria Lymph node involved 0/0 | B |
| C3 | 54 | Rectum | Abdomino-perineal resection | 6 cm diam. into M. propria Lymph node involved 1/6 | C |
| C4 | 58 | Sigmoid 35 cm from anus | Resection | 1 × 1.5 cm diam. into submucosa Lymph node involved 1/6 | C |
| C5 | 61 | Mid transverse colon | Resection | Polypoid. No invasion. Lymph node involved 0 | A |

bowel disease. In addition, a proportion of men were found to have perianal disease or diverticular disease although the significance of these as causes of occult blood loss is dubious. The remaining so-called false positive cases were not investigated for upper gastrointestinal disease, prior consent not having been obtained. No patient had reported gastro-intestinal symptoms or rectal bleeding and none had anaemia.

Table III shows the age at diagnosis in the 17 men in whom adenomatous polyps were found and the size of the largest polyp. One man had 3 polyps, 6 men had 2 and 10 had only 1 polyp. Twelve patients had polyps >1 cm in diameter. All except 1 polyp were removed by snare polypectomy. The exception had a small (0.4 cm) polyp in an area of diverticular disease with a tortuous narrow sigmoid colon which, in spite of two attempts, could not be successfully snared.

**Table III** Diameter[a] of adenomatous polyps and age at diagnosis

| *Age range—years* | *No.* | *<1 cm* | *1–2 cm* | *>2 cm* |
|---|---|---|---|---|
| Under 46 | 2 | 0 | 1 | 1 |
| 46–50 | 5 | 2 | 3 | 0 |
| 51–55 | 5 | 1 | 1 | 3 |
| 56–60 | 3 | 2 | 1 | 0 |
| 61–65 | 2 | 0 | 1 | 1 |
| All | 17 | 5 | 7 | 5 |

[a]Where more than one polyp was present, the diameter of the largest is given.

Surgical resection of the area was carried out without complication. All the polyps and 4/5 cancers were found distal to the splenic flexure. One large malignant polyp was situated in the mid-transverse colon. The mean age at diagnosis of the 17 polyps was 52.6 years and of the 5 cancers 58.8 years. It may reasonably be speculated that removal of the benign lesions may be associated with prevention of malignant disease later. The predictive value of the screening test is improved by excluding individuals under 46 years of age (Table IV). The detection rate in the 46–65 years age range is 1 in 100 for adenomatous polyps and 1 in 300 for cancer in the population screened.

In the 22 men in whom polyps and cancers were found, digital examination of the rectum revealed two cancers. At flexible sigmoidoscopy 13 polyps and 4 cancers were visible and on barium enema examination 8 polyps were noted, but 3 of these had not been visible at sigmoidoscopy. Colonoscopy revealed one polyp and one cancer not visible within the range of the flexible sigmoidoscopy nor on barium enema. The cancer was not seen on a single contrast examination but was visible in the transverse colon when the double contrast technique was used after colonoscopy.

### Discussion

The decision to use Fecatest, a more sensitive Guaiac test for faecal occult blood than the previously more popular Haemoccult, was prompted by an earlier finding of Fecatest positive

**Table IV** Screening for colorectal cancer in the United Kingdom: Details and analysis of studies

| *Reference* | *Population* | *Age range* | *Guaiac test* | *Positive tests no. screened* | *Percentage positive* | *CA+polyp (CA)* | *CA+polyp(CA) %/no. screened* | *CA+polyp(CA) %/no. F.O.B. positive. (predictive value)* |
|---|---|---|---|---|---|---|---|---|
| Hardcastle & Balfour (1980) | General practice | >45 | H* | 27/713 | 3.8 | 6 (2) | 0.84 (0.22) | 22.2 (7.4) |
| Farrands *et al.* (1981) | General practice | >40 | H* | 124/2439 | 5.0 | 12 (4) | 0.49 (0.16) | 9.6 (3.2) |
| Million *et al.* (1982) | General practice | >45 | H* | 37/1646 | 2.3 | 7 (2) | 0.43 (0.12) | 18.9 (5.4) |
| Lee (1983) | Factory based | 40–65 | F** | 140/2420 | 5.8 | 22 (5) | 0.9 (0.21) | 15.7 (3.6) |
| Lee (1983) | Factory based | 46–65 | F** | 92/1575 | 5.8 | 20 (5) | 1.27 (0.32) | 21.7 (5.4) |

H*—Haemoccult.
F**—Fecatest.

Haemoccult negative colorectal cancers (Lee & Costello 1982). Several reports indicate that an undetermined number of colorectal cancers are Haemoccult negative (Griffith *et al.*, 1981; Ribet *et al.*, 1980; Gnauck, 1980) and a false negative rate of 76% for rectosigmoid polyps has been reported (Winawer *et al.*, 1977). Positive Haemoccult tests can convert to negative after a few days' storage (Fleisher *et al.*, 1977; Heinrich *et al.*, 1980) and this is relevant where participants send in their specimens by post. The positivity rate for Haemoccult can be increased by rehydration but some loss of specificity may occur (Fleisher *et al.*, 1977).

Although the majority of studies to date involve Haemoccult, there have been suggestions that a more sensitive test would be useful (Vellacott *et al.*, 1981; Williams *et al.*, 1982). The use of Fecatest in this study has been associated with higher detection rates for colorectal cancer compared with other U.K. series based on general practice populations without a lowering of the predictive value of the basic screening test (Table IV).

Using a more sensitive test may result in an increase in false positivity and one problem here relates to the possibility that upper gastro-intestinal disease may be responsible for positive faecal occult blood tests in asymptomatic patients. With a sensitive test such as Fecatest the importance of proper dietary restriction should be stressed. This requires explanation to participants and a strong motivation to co-operate. It may be that these requirements are more likely to be met in screening a relatively young population, such as that based on place of work, rather than in surveys based in general practice, which usually include a higher proportion of elderly participants.

Although flexible sigmoidoscopy is useful in surveillance it cannot replace double contrast barium enema or colonoscopy since up to 50% of lesions may be beyond the range of the instruments. In this series 17/22 cancers and polyps (77%) were visible on flexible sigmoidoscopy. This high yield may reflect the relatively young population involved, since the incidence of right sided lesions increases with age (Slater *et al.*, 1982). In one large series, 95% of malignant polyps were located distal to the splenic flexure (Appel, 1982) and in another large colonoscopic study only 34% of polyps and 36% of cancers were located beyond the range of the flexible sigmoidoscope (Tedesco *et al.*, 1980). Clearly examination of the whole colon in screening programmes is needed, but it seems that the diagnostic return is likely to be less in the right colon, the younger the population studied. Fork (1981) has compared the diagnostic accuracy of double contrast barium enema as assessed by colonoscopy and concluded that when both techniques are used only few polyps will be missed. The need for careful radiographic techniques is stressed.

Our finding of a co-operation rate of 45% in Factory A compared with 22% in Factory B coincides with previous observation that acceptance is more likely in groups attending for regular medical examination (Glober & Peskoe, 1974; Winawer *et al.*, 1977). Hardcastle *et al.* (1980) noted that in a general practice population compliance was highest in the age range 50–65 years declining sharply over 75 years of age. There was an overall compliance rate of 45% in their study compared with 27% in the "Frome experience" (Farrands *et al.*, 1981) and 28% in Salford (Million *et al.*, 1982). If screening programmes are demonstrated to be beneficial in terms of early diagnosis and prevention of colorectal cancer, more research is needed into optimum recruitment procedures (Halper *et al.*, 1980; Hardcastle *et al.*, 1983).

The co-operation of Dr Robin Goodfellow, British Nuclear Fuels Limited, and the late Dr Geoffrey Purnell, British Aerospace, is gratefully acknowledged along with their nursing and technical staff. Mrs M. Hardman and Mrs J. Hodskinson with the co-operation of the nursing staff in the Department of Gastroenterology, Blackpool Victoria Hospital, gave invaluable help in the planning, execution and interpretation of the study, which was supported in part by a grant from the Cancer Endowment Fund of Blackpool Victoria Hospital.

### References

ADLERCREUTZ, H., LIEWENDAHL, K. & VIRKOLA, P. (1978). Evaluation of Fecatest, a new Guaiac test for occult blood in faeces. *Clin. Chem.*, **24**, 756.

APPEL, M.F. (1982). Distribution of malignant polyps in the colon. *Dis. Colon Rectum*, **25**, 427.

BRALOW, S.P. (1979). *Colorectal Cancer-Screening Programme in Serasota, Florida.* Symposium on colorectal cancer, New York.

BUSSEY, H.J.R. (1963). The long-term results of surgical treatment of cancer of the rectum. *Proc. R. Soc. Med.*, **56**, 494.

DALES, L.G., FRIEDMAN, G.D., RAMCHARAN, S. & 4 others. (1973). Multiphasic check-up evaluation study 3. Outpatient clinic utilization, hospitalization and mortality experience after seven years. *Prevent. Med.*, **2**, 221.

FARRANDS, P.A., GRIFFITHS, R.L. & BRITTON, D.C. (1981). The Frome Experiment—value of screening for colorectal cancer. *Lancet*, **i**, 1231.

FLEISHER, M., SCHWARTZ, K.K. & WINAWER, S.J. (1977). The false-negative Haemoccult test. *Gastroenterology*, **72**, 782.

FORK, F.T. (1981). Double contrast enema and colonoscopy in polyp detection. *Gut*, **22**, 971.

FRÜHMORGEN, P. & DEMLING, L. (1980). Early detection of colorectal cancer with a modified Guaiac test—a screening examination in 6000 humans. In: *Colorectal Cancer: Prevention, Epidemiology and Screening* (Eds. Winawer *et al.*). New York: Raven Press, p. 311.

GILBERTSEN, V.A. (1973). Proctosigmoidoscopy and polypectomy in reducing the incidence of rectal cancer. *Cancer*, **34**, 936.

GILL, P.G. & MORRIS, P.J. (1978). The survival of patients with colorectal cancer treated in a regional hospital. *Br. J. Surg.*, **65**, 17.

GLOBER, G.A. & PESKOE, S.M. (1974). Outpatient screening for gastrointestinal lesions using Guaiac impregnated slides. *Dig. Dis. Sci.*, **19**, 399.

GNAUCK, R. (1980). Occult blood tests. *Lancet*, **i**, 822 (Letter).

GREEGOR, D.H. (1971). Occult blood testing for detection of asymptomatic colon cancer. *Cancer*, **28**, 841.

GRIFFITH, C.D.M., TURNER, D.J. & SAUNDERS, J.H. (1981). False-negative results of Haemoccult. *Br. Med. J.*, **ii**, 472.

GRINNELL, R.S. & LANE, N. (1958). Benign and malignant adenomatous polyps and papillary adenomas of the colon and rectum. An analysis of 1856 tumours in 1335 patients. *Int. Abst. Surg.*, **106**, 519.

HALPER, M.S., WINAWER, S., BRODY, R.S., ANDREWS, M., ROTH, D. & BURTON, G. (1980). Issues of patient compliance. In: *Colorectal Cancer: Prevention, Epidemiology and Screening* (Eds. Winawer *et al.*), New York: Raven Press, p. 299.

HARDCASTLE, J.D., FARRANDS, P.A., BALFOUR, T.W., CHAMBERLAIN, J., AMAR, S.S. & SHELDON, M.G. (1983). Controlled trial of faecal occult blood testing in the detection of colorectal cancer. *Lancet*, **ii**, 1.

HARDCASTLE, J.D., BALFOUR, T.W. & AMAR, S.S. (1980). Screening for symptomless colorectal cancer by testing for occult blood in General Practice. *Lancet*, **i**, 791.

HEINRICH, H.C. & ICAGIC, F. (1980). Comparative studies on the *in vivo* sensitivity of four commercial pseudo-peroxidase based faecal occult blood tests in relation to actual blood losses as calculated from measured whole body—59Fe elimination rate. *Klin. Wochenschr.*, **58**, 1283.

HERTZ, R.E., DEDDIS, M.R. & DAY, E. (1960). Value of periodic examination in detecting cancer of the rectum and colon. *Postgrad. Med.*, **27**, 290.

LEE, F.I. & COSTELLO, F.T. (1982). Assessment of Fecatest and Haemoccult for faecal occult blood testing. *Br. Med. J.*, **ii**, 285, 938.

MILLER, S.F. & KNIGHT, A.R. (1977). The early detection of colorectal cancer. *Cancer*, **40**, 945.

MILLION, R., HOWARTH, J., TURNBERG, E. & TURNBERG, L.A. (1982). Faecal occult blood testing for colorectal cancer in general practice. *Practitioner*, **226**, 659.

MORSON, B.C. (1976). Genesis of colorectal cancer. *Clin. Gastroenterol.*, **5**, 505.

OFFICE OF POPULATION. CENSUSES AND SURVEYS. (1981). *Cancer Statistics: Registrations 1975*. Series MB1, **5**, London, HMSO.

RIBET, A., FREXINOS, J., ESCOURROU, J. & DELPU, J. (1980). Occult blood tests and colorectal tumours. *Lancet*, **i**, 417.

SLATER, G., PAPATESTAS, A.E., TARTTER, P.I., MULVIHILL, M. & AUFSES, A.H. (1982). Age distribution of right- and left-sided colorectal cancers. *Am. J. Gastroenterol.*, **77**, 63.

TEDESCO, F.J., WAYE, J.D., AVELLA, J.R. & VILLALOBOS, M.M. (1980). Diagnostic implications of the spatial distribution of colonic mass lesions (polyps and cancers). *Gastrointest. Endosc.*, **26**, 95.

VELLACOTT, K.D., BALDWIN, R.W. & HARDCASTLE, J.D. (1981). An immunofluorescent test for faecal occult blood. *Lancet*, **i**, 18.

WILLIAMS, J.A.R., HUNTER, R., SMITH, M., COLES, M.O., HUBERT, T.W. & THOMAS, D.W. (1983). Evaluation of an immunological test for occult bleeding from colorectal neoplasia. *Aust. NZ Surg.*, **52**, 617.

WINAWER, S.J., ANDREWS, M., FLEHINGER, B., SHERLOCK, P., SCHOTTENFELD, D. & MILLER, D.G. (1980). Progress report on controlled trial of faecal occult blood testing for the detection of colorectal neoplasia. *Cancer*, **45**, 2959.

WINAWER, S.J., MILLER, D.G., SCHOTTENFIELD, D. & 5 others. (1977). Feasibility of faecal occult blood testing for detection of colorectal neoplasia. *Cancer*, (suppl.), **40**, 2616.